# Seelische Gesundheit und psychische Belastungen von Kindern und Jugendlichen in der ersten Welle der COVID-19-Pandemie – Ergebnisse der COPSY-Studie

**DOI:** 10.1007/s00103-021-03291-3

**Published:** 2021-03-01

**Authors:** Ulrike Ravens-Sieberer, Anne Kaman, Christiane Otto, Adekunle Adedeji, Ann-Kathrin Napp, Marcia Becker, Ulrike Blanck-Stellmacher, Constanze Löffler, Robert Schlack, Heike Hölling, Janine Devine, Michael Erhart, Klaus Hurrelmann

**Affiliations:** 1 Zentrum für Psychosoziale Medizin, Klinik für Kinder- und Jugendpsychiatrie, -psychotherapie und -psychosomatik, Universitätsklinikum Hamburg-Eppendorf, Martinistraße 52, 20246 Hamburg, Deutschland; 2 Abteilung für Epidemiologie und Gesundheitsmonitoring, Fachgebiet Psychische Gesundheit, Robert Koch-Institut, Berlin, Deutschland; 3 Alice Salomon Hochschule, Berlin, Deutschland; 4 Apollon Hochschule der Gesundheitswirtschaft, Bremen, Deutschland; 5 Hertie School, Berlin, Deutschland

**Keywords:** Coronavirus, Psychische Gesundheit, Gesundheitsbezogene Lebensqualität, Depressive Symptome, Ängstlichkeit, Coronavirus, Mental health problems, Health-related quality of life, Depressive symptoms, Anxiety

## Abstract

**Hintergrund:**

Die mit der COVID-19-Pandemie einhergehenden Veränderungen und Kontaktbeschränkungen können das psychische Wohlbefinden von Kindern und Jugendlichen beeinflussen.

**Ziel der Arbeit:**

COPSY ist die erste deutschlandweite repräsentative Studie, welche die psychische Gesundheit und Lebensqualität von Kindern und Jugendlichen während der Pandemie untersucht. Die Ergebnisse werden mit denen der repräsentativen longitudinalen BELLA-Studie aus der Zeit vor der Pandemie verglichen.

**Material und Methoden:**

Vom 26.05. bis zum 10.06.2020 wurden *n* = 1586 Eltern mit 7‑ bis 17-jährigen Kindern und Jugendlichen, von denen *n* = 1040 11- bis 17-Jährige auch Selbstangaben machten, befragt. Dabei wurden international etablierte Instrumente zur Erfassung von gesundheitsbezogener Lebensqualität, psychischen Auffälligkeiten, Ängstlichkeit und depressiven Symptomen eingesetzt. Die Daten wurden mittels deskriptiver Statistiken und bivariater Tests ausgewertet.

**Ergebnisse:**

71 % der Kinder und Jugendlichen und 75 % der Eltern fühlten sich durch die erste Welle der Pandemie belastet. Im Vergleich zu der Zeit vor der Pandemie gaben die Kinder und Jugendlichen eine geminderte Lebensqualität an, der Anteil von Kindern und Jugendlichen mit psychischen Auffälligkeiten hat sich in etwa verdoppelt und ihr Gesundheitsverhalten hat sich verschlechtert. Sozial benachteiligte Kinder erlebten die Belastungen durch die Pandemie besonders stark. Zwei Drittel der Eltern wünschten sich Unterstützung im Umgang mit ihrem Kind.

**Diskussion:**

Die COVID-19-Pandemie führt zu einer psychischen Gesundheitsgefährdung der Kinder und Jugendlichen, auf die präventiv mit niedrigschwelligen und zielgruppenspezifischen Angeboten in der Schule, in der ärztlichen Praxis und in der Gesellschaft im Sinne des Kinderschutzes reagiert werden sollte.

## Abstract (en)

**Background:**

The drastic changes during the COVID-19 pandemic may have a negative impact on the psychological wellbeing of children and adolescents.

**Objectives:**

COPSY is the first national, representative German study to examine mental health and quality of life of children and adolescents during the pandemic. Results are compared with data of the representative longitudinal BELLA study conducted before the pandemic.

**Materials and methods:**

Internationally established instruments for measuring health-related quality of life and mental health (including anxiety and depressive symptoms) were administered to *n* = 1586 parents with 7‑ to 17-year-old children and adolescents, of whom *n* = 1040 11- to 17-year-olds also provided self-reports, from 26 May to 10 June 2020. Data were analyzed using descriptive statistics and bivariate tests.

**Results:**

Seventy-one percent of the children and adolescents and 75% of the parents felt burdened by the first wave of the COVID-19 pandemic. Compared to the time before the pandemic, the children and adolescents reported a lower health-related quality of life, the percentage of children and adolescents with mental health problems almost doubled, and their health behavior worsened. Socially disadvantaged children felt particularly burdened by the COVID-19 pandemic. Two-thirds of the parents would like to receive support in coping with their child during the pandemic.

**Conclusions:**

The COVID-19 pandemic poses a mental health risk to children and adolescents. Schools, doctors, and society are called to react by providing low-threshold and target-group-specific prevention and mental health promotion programs.

## Einleitung

Durch die Coronavirus-Krankheit-2019(COVID-19)-Pandemie und die damit einhergehende Implementierung von Infektionsschutzmaßnahmen wie Quarantäne und Kontaktbeschränkungen kam es zu massiven Veränderungen des täglichen Lebens. Innerhalb weniger Tage hatte sich seit März 2020 das Leben von 13 Mio. Kindern und Jugendlichen in Deutschland schlagartig verändert. Schulen und Kitas wurden geschlossen, Spielplätze waren gesperrt, der Kontakt zu Freunden und Angehörigen war eingeschränkt und die Kinder und Jugendlichen konnten ihren gewohnten Freizeitaktivitäten nicht mehr nachgehen.

Diese abrupten Veränderungen können für Kinder und Jugendliche kritische Lebensereignisse sein. Aus der Forschungsliteratur ist bekannt, dass kritische Lebensereignisse zu psychischen Problemen bei Kindern und Jugendlichen führen können [1, 2]. Ein Rapid-Review (schnelle Evidenzsynthese) fand 7 präpandemische Studien, die beschreiben, dass Quarantäne zu Isolationsgefühlen, Stigmatisierung und Angst führen kann. Als häufigste in dem Zusammenhang auftretende psychische Störungen wurden die akute Belastungsreaktion, Anpassungsstörungen, Trauer und posttraumatische Belastungsstörungen gefunden. Zwei Studien, die während der COVID-19-Pandemie durchgeführt wurden, berichten von Unruhe, Gereiztheit, Anhänglichkeit und Unaufmerksamkeit sowie von einem zunehmenden Medienkonsum bei Kindern und Jugendlichen während der Quarantäne [3, 4].

Nichtrepräsentative Studien aus China zeigen, dass die COVID-19-bedingten Isolations- und Lockdownmaßnahmen mit depressiven Symptomen (23 % bis 44 %) und Angstsymptomen (19 % bis 37 %) bei Kindern einhergehen [5, 6]. Eine Studie aus Indien berichtet über Sorgen (69 %), Hilflosigkeit (66 %) und Angst (62 %) bei Kindern während des Lockdowns [7]. Zusammenhänge zwischen Angst und der COVID-19-Pandemie fanden sich auch in einer aktuellen Studie aus Brasilien [8]. In Studien aus den USA berichten Eltern von einer schlechteren psychischen Gesundheit ihrer Kinder [9, 10] und in einer deutschlandweiten Studie gaben 18 % der Eltern an, dass sich ihre Kinder häufig Sorgen wegen der Coronakrise machen [11]. Nichtrepräsentative Studien aus Spanien und Italien weisen ebenfalls darauf hin, dass Verhaltensprobleme, Reizbarkeit und Einsamkeit bei Kindern und Jugendlichen während der Pandemie zugenommen haben [3, 12]. Die nach unseren Recherchen erste längsschnittliche Studie stammt aus England und belegt, dass depressive Symptome unter Kindern und Jugendlichen während des Lockdowns deutlich zugenommen haben [13].

Während Kinder und Jugendliche vergleichsweise selten an COVID-19 erkranken und meist einen milden oder asymptomatischen Krankheitsverlauf aufweisen [14], legen die oben genannten Studien nahe, dass deren psychische Gesundheit während der Pandemie deutlich gefährdet ist. Kinder und Jugendliche stehen vor entwicklungsbedingten Herausforderungen wie dem Erwerb von Bildung und sozialer Kompetenz [15], der während der COVID-19-Pandemie erschwert ist.

Um die psychische Gesundheit, Lebensqualität und Belastung von Kindern und Jugendlichen während der Pandemie zu erfassen, wurde die COPSY-Studie (**Co**rona und **Psy**che) initiiert. Sie ist unseres Wissens die erste deutschlandweite repräsentative Studie zur psychischen Gesundheit und Lebensqualität von Kindern und Jugendlichen während der COVID-19-Pandemie, in der auch die Kinder und Jugendlichen selbst befragt werden. Die COPSY-Studie nutzt dabei das Befragungsinventar der repräsentativen longitudinalen BELLA-Studie (**Be**fragung zum see**l**ischen Woh**l**befinden und Verh**a**lten), wodurch ein Vergleich der psychischen Gesundheit vor und während der Pandemie möglich ist. Darüber hinaus soll untersucht werden, welche Kinder und Jugendlichen besonders durch die Auswirkungen der COVID-19-Pandemie belastet werden und welche Unterstützung nötig ist.

## Methoden

### Studiendesign und Stichprobe

Die COPSY-Studie wurde in Anlehnung an das Design und die Methodik der repräsentativen longitudinalen BELLA-Kohortenstudie konzipiert. Die BELLA-Studie ist das Modul zur psychischen Gesundheit der Studie zur Gesundheit von Kindern und Jugendlichen in Deutschland (KiGGS), welche seit 2003 in Kooperation mit dem Robert Koch-Institut durchgeführt wird [16, 17]. In der BELLA-Studie wurden Kinder und Jugendliche sowie deren Eltern mittels international etablierter Instrumente zur psychischen Gesundheit und Lebensqualität befragt (nähere Informationen zur BELLA-Studie finden sich bei [17, 18]). Die resultierenden umfangreichen Datensätze wurden als bevölkerungsbasierte Referenzdaten vor der COVID-19-Pandemie zum Vergleich mit der COPSY-Stichprobe genutzt.

Die COPSY-Studie wurde vom 26.05. bis zum 10.06.2020 vom Universitätsklinikum Hamburg-Eppendorf (UKE) in Zusammenarbeit mit der Infratest dimap Gesellschaft für Trend- und Wahlforschung mbH bundesweit durchgeführt. Während dieser Zeit befand sich Deutschland noch unter einem moderaten Lockdown. Erste Schulen und Freizeiteinrichtungen wurden langsam wieder geöffnet und Kontaktbeschränkungen wurden gelockert. Kinder und Jugendliche sowie deren Eltern wurden zu den Auswirkungen der ersten Welle der COVID-19-Pandemie und der damit verbundenen Maßnahmen auf die psychische Gesundheit und Lebensqualität befragt.

Insgesamt wurden *n* = 3597 Familien mit Kindern und Jugendlichen im Alter von 7 bis 17 Jahren zur Teilnahme an der COPSY-Studie eingeladen. Die Familien wurden kontaktiert, über die Studie informiert und um ihre Einwilligung zur Teilnahme gebeten. Insgesamt haben *n* = 1586 Eltern von 7‑ bis 17-jährigen Kindern und Jugendlichen sowie *n* = 1040 Kinder und Jugendliche im Alter von 11 bis 17 Jahren an der Studie teilgenommen und den Fragebogen online ausgefüllt. Es wurde ein Gewichtungsfaktor berechnet, damit die Stichprobe in den wesentlichen Merkmalen der Struktur der Grundgesamtheit der Eltern von Kindern im Alter von 7 bis 17 Jahren in Deutschland laut aktuellem Mikrozensus (2018) entspricht. Die COPSY-Studie wurde vorab von der Lokalen Psychologischen Ethikkommission am Zentrum für Psychosoziale Medizin (LPEK) des UKE ethisch und fachrechtlich beraten (LPEK-0151) sowie vom Datenschutzbeauftragten des UKE begleitet.

### Erhebungsverfahren

Gemäß den Empfehlungen des *International Consortium for Health Outcomes Measurement* (ICHOM; [19]) wurden international etablierte Fragebögen eingesetzt, um die gesundheitsbezogene Lebensqualität (KIDSCREEN-10-Index [20]), psychische Auffälligkeiten (Strenghts and Difficulties Questionnaire, SDQ [21]), generalisierte Ängstlichkeit (Screen for Child Anxiety Related Emotional Disorders, SCARED [22]) und depressive Symptome (Center for Epidemiological Studies Depression Scale for Children, CES-DC [23] und Patient Health Questionnaire, PHQ [24]) zu erheben.

Darüber hinaus wurde das Belastungserleben der Kinder und Jugendlichen sowie von deren Eltern mithilfe eines selbst entwickelten Items erfasst („Wie belastend waren Veränderungen im Zusammenhang mit der Corona-Krise für Sie/dich insgesamt?“; 5‑stufige Antwortskala von 1 = *gar nicht belastend* bis 5 = *äußerst belastend*). Des Weiteren wurden folgende Aspekte des Gesundheitsverhaltens der Kinder und Jugendlichen erfasst. Der Medienkonsum wurde anhand von 2 selbst entwickelten Items erfragt („Wie viele Stunden verbringst du zurzeit insgesamt pro Tag mit Computer, Smartphone, Tablets, Spielekonsole (d. h. digitalen Medien) für schulische Aufgaben/für private Angelegenheiten?“ (Angaben in Stunden) sowie „Und ist das im Vergleich zur Zeit vor der Corona-Krise …?“ (Antwortoptionen: 1 = *viel weniger* bis 5 = *viel mehr*)). Die körperliche Aktivität wurde unter Nutzung eines Items aus der internationalen HBSC-Studie erhoben („An wie vielen Tagen hast du dich in der letzten Woche für mindestens 60 Minuten körperlich angestrengt?“), welches auf einer 8‑stufigen Skala beantwortet wurde (1 = *0 Tage* bis 8 = *7 Tage*). Das Ernährungsverhalten der Kinder und Jugendlichen wurde mithilfe eines selbst entwickelten Items erfasst („Wenn du nochmals an die Zeit vor der Corona-Krise denkst: Hast du in der letzten Woche weniger, gleich viel oder mehr Süßigkeiten als vor der Corona-Krise gegessen?“; Antwortoptionen: 1 = *viel weniger* bis 5 = *viel mehr*).

Des Weiteren wurden die Eltern mithilfe von 3 selbst entwickelten Items zu ihrem Unterstützungsbedarf befragt („Würden Sie sich im Umgang mit Ihrem Kind während der Corona-Krise Unterstützung wünschen?“ (Antwortoptionen: 1 = *nein, nie* bis 4 = *ja, immer*), „In welchen Bereichen hätten Sie gern Unterstützung?“ (Antwortoptionen siehe Abb. 4) sowie „Wie möchten Sie diese Unterstützung bekommen?“ (Antwortoptionen: 1 = *Schriftliches Online-Material*, 2 = *Online-Videos*, 3 = *Fernsehsendungen*, 4 = *Podcasts*, 5 = *Telefonische Hotline*, 6 = *Online-Hotline*, 7 = *Persönliche Unterstützung von anderen Eltern (online)*, 8 = *Unterstützung von Freunden, Bekannten oder der Familie*, 9 = *Persönliche Unterstützung von Experten (online oder telefonisch)*, 10 = *Persönliches Gespräch mit einem Experten*, 11 = *Online-Selbsthilfegruppe für Eltern*, 12 = *Schule/Lehrer*, 13 = *Sonstiges*)).

### Statistische Analysen

Die Datenauswertung erfolgte mithilfe deskriptiver Statistiken (absolute und relative Häufigkeiten, Mittelwerte und Standardabweichungen) sowie bivariater Tests (Chi-Quadrat-Tests). Alle Analysen wurden mit SPSS Version 26 durchgeführt. Signifikante Unterschiede zwischen Gruppen wurden bei einem Signifikanzniveau von *p* < 0,05 angenommen. Es wurden keine statistischen Adjustierungen für Alter und Geschlecht vorgenommen, da die Alters- und Geschlechtsstruktur der untersuchten Kollektive aufgrund der Gewichtung auf die Bevölkerungsverhältnisse als vergleichbar angesehen werden kann. Auch für Subgruppenanalysen (Migrationshintergrund, Bildungsstatus) erfolgte keine Adjustierung, da sich diese Gruppen in A‑priori-Analysen nicht nennenswert in ihrer Alters- und Geschlechtsstruktur unterschieden (Ergebnisse nicht berichtet). Zum Vergleich der T‑Werte, die aus dem Eltern- und Selbstbericht des KIDSCREEN-10-Index resultieren, wurde ein gepaarter t‑Test durchgeführt. Die zugehörige Interraterreliabilität wurde mithilfe der Intraklassenkorrelation geprüft (einzelne Rater, absolute Übereinstimmung).

## Ergebnisse

Insgesamt nahmen *n* = 1586 Familien mit Kindern im Alter von 7 bis 17 Jahren (*M* = 12,25; *SD* = 3,30; 50,0 % weiblich) an der COPSY-Studie teil. Das durchschnittliche Alter der Eltern betrug 43,99 Jahre (*SD* = 7,36). Die Mehrheit der Kinder und Jugendlichen hatte keinen Migrationshintergrund (84,0 %). Die meisten Eltern hatten ein mittleres Bildungsniveau (55,7 %), waren verheiratet (69,2 %) und in Vollzeit angestellt (51,7 %). Weitere Charakteristika der Studienpopulation sind in Tab. 1 beschrieben.

**Table 1:** 

	Eltern von Kindern im Alter von 7 bis 17 Jahren (*n* = 1586)		Kinder und Jugendliche im Alter von 11 bis 17 Jahren (*n* = 1040)	
	*n* (%)	*M (SD)*	*n* (%)	*M (SD)*
*Alter des Kindes*	–	12,25 (3,30)	–	14,33 (1,86)
*Geschlecht des Kindes*				
Männlich	791 (49,9)	–	508 (48,8)	–
Weiblich	793 (50,0)	–	531 (51,1)	–
Divers	1 (0,1)	–	1 (0,1)	–
Keine Angabe	1 (0,1)	–	–	–
*Alter der Eltern*	–	43,99 (7,36)	–	46,28 (6,74)
*Migrationshintergrund der Kinder*				
Nein	1332 (84,0)	–	879 (84,5)	–
Ja	254 (16,0)	–	161 (15,5)	–
*Elterliche Bildung*^*a*^				
Niedrig	288 (18,2)	–	192 (18,5)	–
Mittel	884 (55,7)	–	548 (52,7)	–
Hoch	383 (24,1)	–	277 (26,6)	–
Keine Angabe	31 (2,0)	–	23 (2,2)	–
*Familienstand der Eltern*				
Ledig	140 (8,8)	–	87 (8,4)	–
Verheiratet	1097 (69,2)	–	717 (68,9)	–
In einer festen Beziehung	216 (13,6)	–	125 (12,0)	–
In einer eingetragenen Lebenspartnerschaft	13 (0,8)	–	8 (0,8)	–
Geschieden	108 (6,8)	–	92 (8,8)	–
Verwitwet	12 (0,8)	–	11 (1,1)	–
*Berufstätigkeit der Eltern*				
Angestellt in Vollzeit	820 (51,7)	–	561 (53,9)	–
Angestellt in Teilzeit	453 (28,6)	–	286 (27,5)	–
Selbstständig	67 (4,2)	–	49 (4,7)	–
Anderes Beschäftigungsverhältnis	32 (2,0)	–	22 (2,1)	–
Hausfrau/Hausmann	109 (6,9)	–	61 (5,9)	–
Rentner/Pensionär	34 (2,1)	–	27 (2,6)	–
In Elternzeit	29 (1,8)	–	7 (0,7)	–
Nicht berufstätig	42 (2,6)	–	27 (2,6)	–

*M* Mittelwert, *SD* Standardabweichung

^a^Die Differenzierung in Eltern mit niedrigem, mittlerem und hohem Bildungsniveau erfolgte anhand der international etablierten CASMIN-Klassifikation (Comparative Analysis of Social Mobility in Industrial Nations)

### Belastungserleben in der ersten Welle der COVID-19-Pandemie

Insgesamt fühlten sich 70,7 % der Kinder und Jugendlichen und 75,4 % der Eltern durch die Pandemie und die damit einhergehenden Veränderungen belastet. Die Kinder und Jugendlichen fühlten sich vor allem dadurch belastet, dass sie das Homeschooling als anstrengend empfanden (64,4 %), weniger Kontakt zu ihren Freunden hatten (82,8 %) und es häufiger Streit in der Familie gab (27,6 %). Drei Viertel der Eltern (79,0 %) empfanden die Veränderung ihrer beruflichen Situation belastend.

### Lebensqualität in der ersten Welle der COVID-19-Pandemie

**Figure 1:**
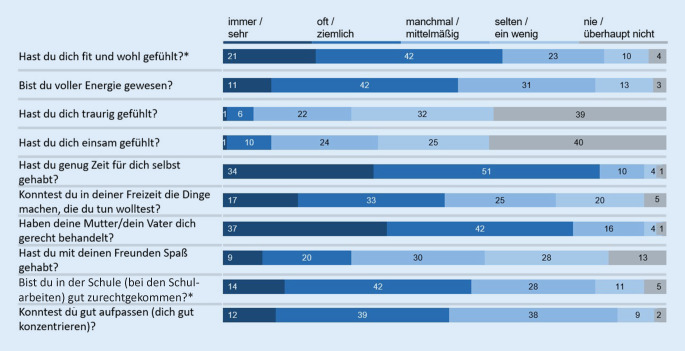

**Figure 2:**
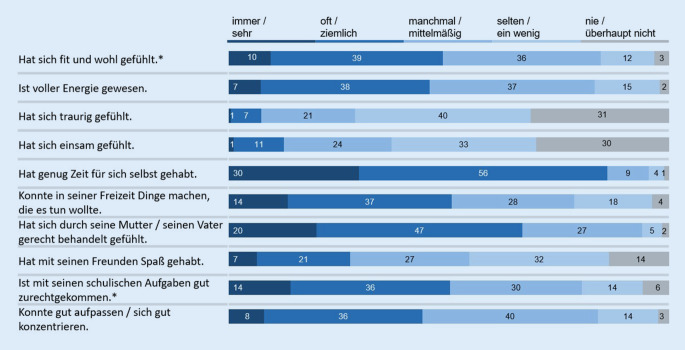

Die Lebensqualität der Kinder und Jugendlichen – gemessen mit dem KIDSCREEN-10-Index – hat sich im Vergleich zu der Zeit vor der COVID-19-Pandemie deutlich verschlechtert: So gaben 40,2 % (*n* = 418 [37,1 %; 43,1 %]) der befragten 11- bis 17-jährigen Kinder und Jugendlichen (*n* = 1040) während der Coronakrise selbst eine geminderte gesundheitsbezogene Lebensqualität an, in der BELLA-Studie vor der Krise war dies nur bei 15,3 % (*n* = 146 [13,0 %; 17,6 %]) der Kinder und Jugendlichen der Fall [25]. Die in der COPSY-Studie befragten Eltern der 7‑ bis 17-Jährigen (*n* = 1586) berichteten für 41,9 % (*n* = 664 [39,5 %; 44,3 %]) ihrer Kinder eine geminderte Lebensqualität, für 54,9 % (*n* = 870 [52,5 %; 57,4 %]) eine mittlere und für 3,2 % (*n* = 52 [29,7 %; 34,3 %]) eine hohe Lebensqualität. Folgend werden die Verteilungen der Itemantworten zur Lebensqualität aus der COPSY-Studie dargestellt (Abb. 1 gemäß Selbstbericht der 11- bis 17-Jährigen, Abb. 2 gemäß Elternbericht für 7‑ bis 17-Jährige).

Der Mittelwert der Lebensqualität (KIDSCREEN-10-Index) aus dem Elternbericht liegt für 7‑ bis 17-Jährige bei 41,17. Betrachtet man ausschließlich 11- bis 17-Jährige findet sich ein Wert von 42,36 und der entsprechende selbstberichtete Wert liegt signifikant höher bei 45,38 (*p* < 0,001). Der zugehörige Intraklassenkorrelationskoeffizient von 0,72 weist laut Cicchetti [26] auf eine gute Übereinstimmung hin.

### Psychische Auffälligkeiten in der ersten Welle der COVID-19-Pandemie

Die Prävalenz für psychische Auffälligkeiten stieg von 17,6 % (*n* = 273 [15,7 %; 19,5 %]) vor der COVID-19-Pandemie auf 30,4 % (*n* = 482 [28,1 %; 32,7 %]) während der Krise. Damit wurden während der Pandemie für fast jedes dritte Kind psychische Auffälligkeiten (erhoben mit dem SDQ) berichtet, während vor der Pandemie etwa jedes fünfte Kind betroffen war.

Darüber hinaus berichteten 24,1 % (*n* = 255 [21,9 %; 27,1 %]) der Kinder und Jugendlichen während der COVID-19-Pandemie Symptome einer generalisierten Angststörung (erhoben mit der entsprechenden Subskala des SCARED), vor der Krise war dies nur bei 14,9 % (*n* = 198 [13,0 %; 16,8 %]) der Fall [25]. Die Kinder und Jugendlichen gaben während der Pandemie für sieben Items signifikant höhere Ängstlichkeitswerte als vor der Pandemie an, allerdings war die Stärke der gefundenen Unterschiede klein (Tab. 2).

**Table 2:** 

		BELLA Studie (*n* = 1333)		COPSY-Studie (*n* = 1040)		Teststatistik			
		„trifft genau oder häufig zu“		„trifft genau oder häufig zu“					
	Items zur Erfassung generalisierter Ängstlichkeit (SCARED-D)	*n*	% [95 %-Konfidenzintervall]	*n*	% [95 %-Konfidenzintervall]	Chi^2^	Df	*p*‑Wert	Effektstärke ϕ
1	Ich mache mir Sorgen darüber, ob andere Menschen mich mögen	70	5,3 % [4,1 %; 6,4 %]	136	13,1 % [11,0 %; 15,2 %]	43,13	1	< 0,001	**0,14**
2	Ich bin nervös	62	4,7 % [3,5 %; 5,8 %]	49	4,7 % [3,4 %; 6,0 %]	< 0,01	1	0,945	*–*
3	Ich mache mir Sorgen, ob ich genauso gut bin wie andere Kinder	54	4,1 % [3 %; 5,1 %]	140	13,5 % [11,4 %; 15,5 %]	68,76	1	< 0,001	**0,17**
4	Ich mache mir Sorgen, ob alles gut läuft	114	8,6 % [7,1 %; 10,1 %]	150	14,4 % [12,3 %; 16,6 %]	20,31	1	< 0,001	0,09
5	Ich bin jemand, der sich viele Sorgen macht	130	9,8 % [8,2 %; 11,3 %]	133	12,8 % [10,8 %; 14,8 %]	5,46	1	0,019	0,05
6	Andere sagen mir, dass ich mir zu viele Sorgen mache	61	4,6 % [3,5 %; 5,7 %]	115	11,1 % [9,2 %; 13,0 %]	35,74	1	< 0,001	**0,12**
7	Ich mache mir Sorgen darüber, was in der Zukunft geschehen wird	169	12,7 % [10,9 %; 14,5 %]	140	13,5 % [11,4 %; 15,6 %]	0,32	1	0,574	*–*
8	Ich bin unsicher, ob ich meine Sache gut mache	71	5,3 % [4,1 %; 6,5 %]	132	12,7 % [10,7 %; 14,8 %]	40,52	1	< 0,001	**0,13**
9	Ich mache mir Sorgen über Dinge, die bereits geschehen sind	51	3,8 % [2,8 %; 4,9 %]	92	8,9 % [7,1 %; 10,6 %]	26,00	1	< 0,001	**0,10**

Angegeben ist jeweils die Anzahl/der Anteil von Kindern und Jugendlichen, die *trifft genau oder häufig zu* angegeben haben (Antwortoptionen: 0 = *trifft nicht oder fast nie zu*, 1 = *trifft manchmal oder etwas zu*, 2 = *trifft genau oder häufig zu*). Effektstärken, die auf kleine Effekte hinweisen, sind fett gedruckt (die verbleibenden Effekte für Items 4 und 5 sind aufgrund der geringen Effektstärke zu vernachlässigen). Die Teststatistik zeigt Resultate von Chi^2^-Tests zum Vergleich der Items über beide Studien (Vierfeldertafel; hierfür wurden je Item und je Studie 2 Gruppen gebildet (Antwortoption 0 versus 1 und 2))

Im Hinblick auf die Häufigkeit depressiver Symptome ergab sich bei der Analyse der Summenwerte über die eingesetzten Items des CES-DC kein interpretierbarer Unterschied im Vergleich zum Zeitraum vor der Pandemie (*p* > 0,05 [25]). Gemäß dem PHQ‑2 berichteten lediglich 11,1 % (*n* = 115 [9,2 %; 13,0 %]) der 11- bis 17-Jährigen, beinahe jeden Tag bzw. an mehr als der Hälfte der Tage *wenig Interesse oder Freude an ihren Tätigkeiten* gehabt zu haben; 47,3 % der Befragten gaben dies für einzelne Tage an (*n* = 492 [44,3 %; 50,3 %]; überhaupt nicht: 41,6 % (*n* = 433 [38,6 %; 44,6 %])). Ein Anteil von 6,6 % (*n* = 67 [5,1 %; 8,1 %]) der Kinder und Jugendlichen erlebte beinahe jeden Tag bzw. an mehr als der Hälfte der Tage *Niedergeschlagenheit, Schwermut oder Hoffnungslosigkeit*, 20,0 % (*n* = 208 [17,6 %; 22,4 %]) erlebten dies nur an einzelnen Tagen (überhaupt nicht: 73,5 % (*n* = 764 [70,8 %; 76,2 %])).

### Risiken

**Figure 3:**
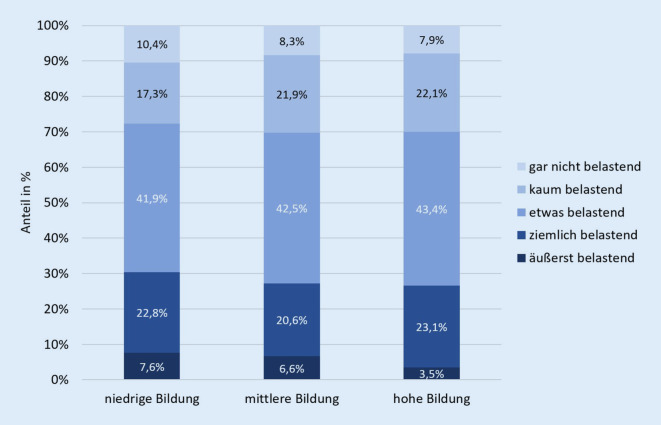

Besonders belastet waren Kinder und Jugendliche, deren Eltern einen niedrigen Bildungsabschluss haben, die einen Migrationshintergrund haben und/oder die auf beengtem Raum leben (< 20 m^2^ Wohnfläche/Person). So berichteten beispielsweise Kinder, deren Eltern einen niedrigen Bildungsabschluss haben, mehr als doppelt so häufig, dass die Veränderungen durch die COVID-19-Pandemie äußerst belastend seien (Abb. 3). Darüber hinaus berichtete ein Drittel (33,2 %) der Kinder, deren Eltern einen niedrigen Bildungsabschluss aufweisen, das Lernen sei im Vergleich viel anstrengender, während nur ein Fünftel (20,4 %) der Kinder mit Eltern, die einen hohen Bildungsabschluss aufweisen, das Lernen viel anstrengender wahrnahmen. Von den Eltern mit Migrationshintergrund berichteten 38,4 %, dass das Lernen für ihre Kinder viel anstrengender geworden sei, was nur 30,5 % der Eltern ohne Migrationshintergrund so empfanden.

### Gesundheitsverhalten in der ersten Welle der COVID-19-Pandemie

Das Gesundheitsverhalten der Kinder und Jugendlichen (erfasst mit den oben beschriebenen Items) hat sich während der Pandemie verschlechtert. So berichteten mehr als 2 Drittel (69,9 %) der Kinder und Jugendlichen eine Zunahme ihres Medienkonsums. Ein Drittel (33,3 %) der Kinder und Jugendlichen verbrachte pro Tag 4 Stunden oder mehr mit der Nutzung von Medien. Darüber hinaus gab ein Fünftel (19,3 %) an, gar keinen Sport zu machen, und ein Viertel (26,3 %) berichtete, etwas bis viel mehr Süßigkeiten als vor der COVID-19-Pandemie zu essen.

### Unterstützungsbedarf

**Figure 4:**
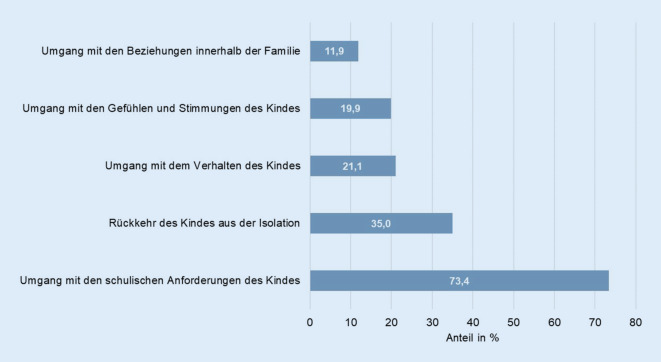

Knapp 2 Drittel (63,0 %) der befragten Eltern wünschten sich im Umgang mit ihrem Kind während der COVID-19-Pandemie Unterstützung. Am häufigsten wünschten sich Eltern Unterstützung bei der Bewältigung der schulischen Anforderungen ihres Kindes, bei der Rückkehr des Kindes aus der Isolation und im Umgang mit dem Verhalten, den Gefühlen und Stimmungen des Kindes (Abb. 4).

Auf die Frage, wie bzw. von wem sich die Eltern diese Unterstützung wünschen, wurden am häufigsten Schule/Lehrer (65,2 %), Freunde/Familie (26,6 %), online/telefonische Unterstützung von Experten (20,2 %), persönliches Gespräch mit Experten (19,2 %) sowie schriftliche Materialien/Ratgeber (19,2 %) genannt.

## Diskussion

Die COPSY-Studie zeigt als erste deutschlandweite repräsentative Studie zur psychischen Gesundheit und Lebensqualität von Kindern und Jugendlichen während der COVID-19-Pandemie, dass sich die Mehrheit der Kinder und Jugendlichen in Deutschland durch die Pandemie belastet fühlt. Im Vergleich zum Zeitraum vor der Pandemie hat sich die Lebensqualität der Kinder und Jugendlichen verschlechtert, Ängstlichkeit und die Häufigkeit psychischer Auffälligkeiten haben zugenommen, die Depressivität ist (noch) nicht signifikant nachweisbar gestiegen, allerdings könnten die Ergebnisse auf Itemebene eine entsprechende Tendenz andeuten.

Die Resultate der COPSY-Studie bestätigen die Ergebnisse bisheriger Studien aus China, Indien, den USA, Spanien und Italien, in denen eine Zunahme von Angst, Stress und anderen Belastungsreaktionen bei Kindern und Jugendlichen während der ersten Welle der Pandemie festgestellt wurde [3, 5–7, 10–12]. Unsere Resultate zu depressiven Symptomen bei Kindern und Jugendlichen stehen in (vermeintlichem) Widerspruch zu den Ergebnissen einer aktuellen britischen Longitudinalstudie, wonach Depressionen bei Kindern und Jugendlichen während der Pandemie bereits zugenommen haben [13]. Aus klinischer Perspektive kann vermutet werden, dass die Pandemie zunächst eher zu Angstreaktionen führte und nun mit einem monatelangen (sozialen) Verstärkerverlust depressive Entwicklungen stärker zutage treten werden. Diese Vermutung gilt es in der COPSY-Folgebefragung, welche während der zweiten Infektionswelle durchgeführt wird, zu untersuchen.

Die vorliegende Arbeit aus der bundesweiten COPSY-Studie beschreibt im Vergleich zu Vorpublikationen [25, 27] Veränderungen in der Lebensqualität, Angst und Depressivität vertiefend (auf der Itemebene). Zudem werden erstmals Daten bezogen auf das zunehmend kritische Gesundheitsverhalten der Kinder und Jugendlichen sowie zum Unterstützungsbedarf der Eltern berichtet. In bisherigen Publikationen zur COPSY-Studie wurde gezeigt, dass während der ersten Welle der Pandemie auch eine Zunahme psychosomatischer Beschwerden und psychischer Auffälligkeiten wie Hyperaktivität und Probleme mit Gleichaltrigen zu verzeichnen ist [25, 27]. Diese Ergebnisse stehen im Einklang mit Ergebnissen von Jiao et al. [4], die bei Schulkindern, die in häuslicher Quarantäne waren, auch vermehrt Hyperaktivität und Probleme mit Gleichaltrigen fanden. Erwähnenswert ist, dass körperliche Bewegung bzw. Sport zu Hause helfen konnten, Hyperaktivität abzumildern. Darüber hinaus fanden Jiao et al. [4], dass die Ängstlichkeit der Eltern einen negativen Einfluss auf die Emotionalität der Kinder hatte. Das Wechselspiel zwischen der psychischen Gesundheit der Kinder und der der Eltern wird vielfach diskutiert.

Zur Einschätzung der Lebensqualität durch verschiedene Beurteiler zeigt eine Übersichtsarbeit von Upton et al. [28], dass Eltern von gesunden Kindern die Lebensqualität ihrer Kinder höher einschätzen als die Kinder selbst; hingegen schätzen Eltern von kranken Kindern die Lebensqualität ihrer Kinder geringer ein als diese selbst. Dies konnte auch in Studien zur Interraterübereinstimmung zwischen Kindern mit ADHS (Aufmerksamkeitsdefizit-Hyperaktivitätsstörung) und deren Eltern nachgewiesen werden [29]. Die Pandemie mit ihren Herausforderungen ist eine kritische Situation, in der Eltern scheinbar ähnlich wie bei vorliegender Erkrankung ihres Kindes, dessen Lebensqualität tendenziell eher geringer als ihr Kind selbst einschätzen. Die gute Übereinstimmung beider Urteile in der COPSY-Studie ist eventuell auf die während der Studiendurchführung geltenden Maßnahmen und die daraus resultierende ausgeprägte räumliche Nähe in den Familien zurückzuführen.

Die COPSY-Studie zeigt, dass sich drei Viertel der Eltern durch berufliche Veränderungen während der Pandemie belastet fühlen und sich mehr Unterstützung wünschen. Aktuelle Studien aus den USA zeigen, dass ein Arbeitsplatzverlust und finanzielle Belastungen sowie Schwierigkeiten, die Kinderbetreuung zu gewährleisten, Risikofaktoren für die psychische Gesundheit der Eltern selbst als auch ihrer Kinder darstellen [9, 10]. Andere aktuelle Studien beschreiben, dass Eltern besonders gestresst sind und hohe Neurotizismuswerte haben, wenn sie jüngere bzw. viele Kinder haben, alleinerziehend sind oder wenn ihre Kinder emotionale, behaviorale oder andere psychische Störungen haben [30, 31]. Diese Eltern sind gefährdet, sich während der Pandemie sehr zu erschöpfen und ein „Burn-out“ zu entwickeln [32]. Dies sollte bei zukünftigen politischen Entscheidungen im Rahmen weiterer Infektionswellen berücksichtigt werden [33].

Nach unserem Kenntnisstand zeigt die COPSY-Studie erstmals, dass sich das Gesundheitsverhalten der Kinder während der Pandemie verschlechtert hat: Der Medienkonsum ist hoch, ein Fünftel der Kinder treibt keinen Sport und ein Drittel isst mehr Süßigkeiten als vor der COVID-19-Pandemie. Aktuelle internationale Studien weisen in eine ähnliche Richtung. Beispielsweise zeigte eine italienische Studie, dass der Medienkonsum von Kindern und Jugendlichen während der Pandemie um 4 Stunden pro Tag zunahm, während die körperliche Aktivität um mehr als 2 Stunden pro Tag abnahm [34]. Ein erhöhter Konsum von Computerspielen während der Pandemie wurde von King et al. [35] beschrieben. Eine frühere Studie zeigt, dass ein verstärkter Medienkonsum auch mit Veränderungen von Essgewohnheiten einhergehen kann und somit das Risiko für Übergewicht und zugehörige Folgeerkrankungen steigen kann [36]. Eine weitere Studie zur Mediensucht bei Kindern während der COVID-19-Pandemie weist darauf hin, dass ein Medienmissbrauch nicht nur Schlafgewohnheiten negativ beeinflussen, sondern sich auch negativ auf die Lebensqualität auswirken kann. Diese Studien lassen vermuten, dass sich die beschriebenen ungünstigen Gesundheitsverhaltensweisen und die Entwicklung psychischer Erkrankungen gegenseitig bedingen und vermutlich verstärken können. Dieses Wechselspiel stellt mittel- bis langfristig ein Gesundheitsrisiko für die Kinder und Jugendlichen dar. Die Entwicklung entsprechender Präventionsmaßnahmen zum Einsatz während dieser bzw. zukünftiger Pandemien ist daher dringend geboten.

Darüber hinaus ist das Ergebnis der COPSY-Studie relevant, dass Streitigkeiten in den Familien zunehmen und öfter eskalieren. In anderen Studien konnte bereits gezeigt werden, dass das Risiko von Kindesmissbrauch und Vernachlässigung in Krisenzeiten steigt [37, 38], sodass UNICEF und der Deutsche Kinderschutzbund zu Recht dringende Unterstützung vom Erziehungs- und Bildungssystem, von Ärzten und Politikern fordern, um Kinder und Jugendliche zu schützen. Bei weiteren Entscheidungen der Regierung sollten daher familienpolitische sowie kinder- und jugendhilferechtliche Perspektiven stärker berücksichtigt werden [38, 39].

Die vorliegende Studie zeigt auch, dass sozial benachteiligte Kinder und Jugendliche besonders stark von den Auswirkungen der COVID-19-Pandemie betroffen sind. Soziale Ungleichheiten in Bezug auf die psychische Gesundheit wurden bereits in zahlreichen Studien belegt [1, 40]. Um diese Ungleichheiten zu verringern, werden flächendeckende, zielgruppenspezifische und niedrigschwellige Angebote der Prävention und Gesundheitsförderung benötigt.

Um die Bewältigung der Krise von Kindern und Jugendlichen zu unterstützen, haben die Bundeszentrale für gesundheitliche Aufklärung (BZgA; [41]) und das Bundesamt für Bevölkerungsschutz und Katastrophenhilfe (BBK; [42]) Empfehlungen zur Unterstützung von Familien veröffentlicht, wie z. B. dass Eltern mit ihren Kindern über die Situation und ihre Sorgen offen sprechen mögen, dass ein strukturierter Tagesablauf mit festen Schlaf- und Essenszeiten Kindern Halt und Sicherheit vermitteln kann und dass Zeit an der frischen Luft und Bewegung helfen können, das Belastungserleben und Risiken für die psychische Gesundheit von Kindern und Jugendlichen abzubauen. Diese und weitere Empfehlungen zur Förderung der psychischen Gesundheit von Kindern und Jugendlichen während der Pandemie finden sich auch zunehmend in wissenschaftlichen Publikationen [43–47].

Die Stärken der vorliegenden Studie liegen im Einsatz international etablierter Fragebögen sowie im Vergleich der Ergebnisse mit der repräsentativen longitudinalen BELLA-Studie aus der Zeit vor der Pandemie. Aufgrund des Querschnittdesigns konnten jedoch keine kausalen Zusammenhänge untersucht werden. Zudem wurden psychische Auffälligkeiten nicht mit klinischen Interviews diagnostiziert, sondern mit Screeningfragebögen erfasst.

Die Ergebnisse der COPSY-Studie, vor allem auch die Ergebnisse zum Unterstützungsbedarf der Eltern, sollten Ärzte/Therapeuten, Lehrer/Erzieher, Eltern und Politiker anregen, die psychische Gesundheit und Belastungen sowie die Bedürfnisse von Kindern und Jugendlichen bei zukünftigen Infektionswellen und Entscheidungen stärker mit in den Blick zu nehmen. Es ist dringend zu empfehlen, belastete Kinder, Jugendliche und Eltern zu unterstützen, um deren psychische Gesundheit zu schützen bzw. aufrechtzuerhalten.
